# Supplementary figures and images for: Extensive remodelling of the cell wall during the development of Staphylococcus aureus bacteraemia
Source: eLife. 2023 Jul 4;12:RP87026. doi: 10.7554/eLife.87026 (PMC10328498; doi:10.7554/eLife.87026)

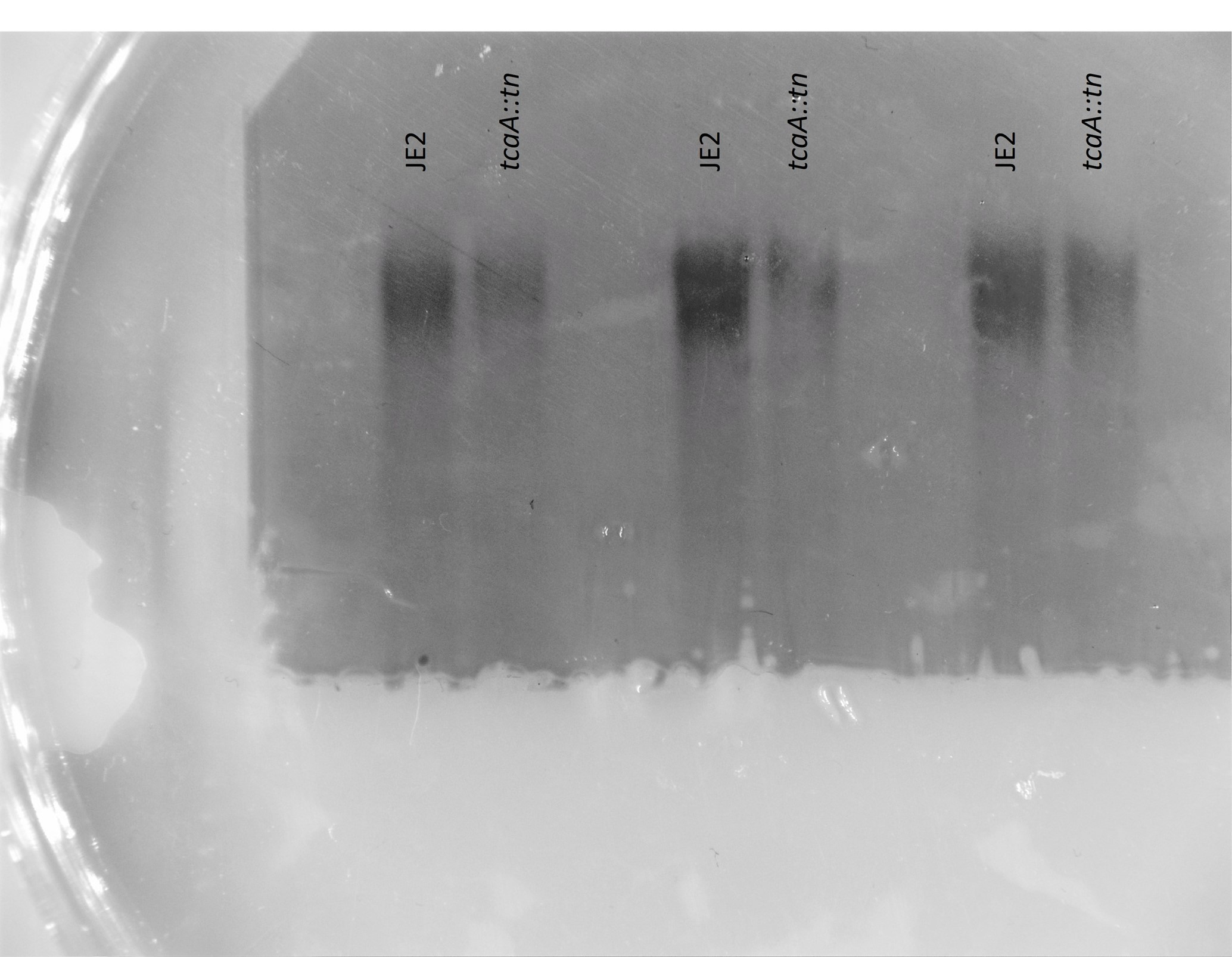

Supplement: Figure 5—source data 1. [file elife-87026-fig5-data1.docx]

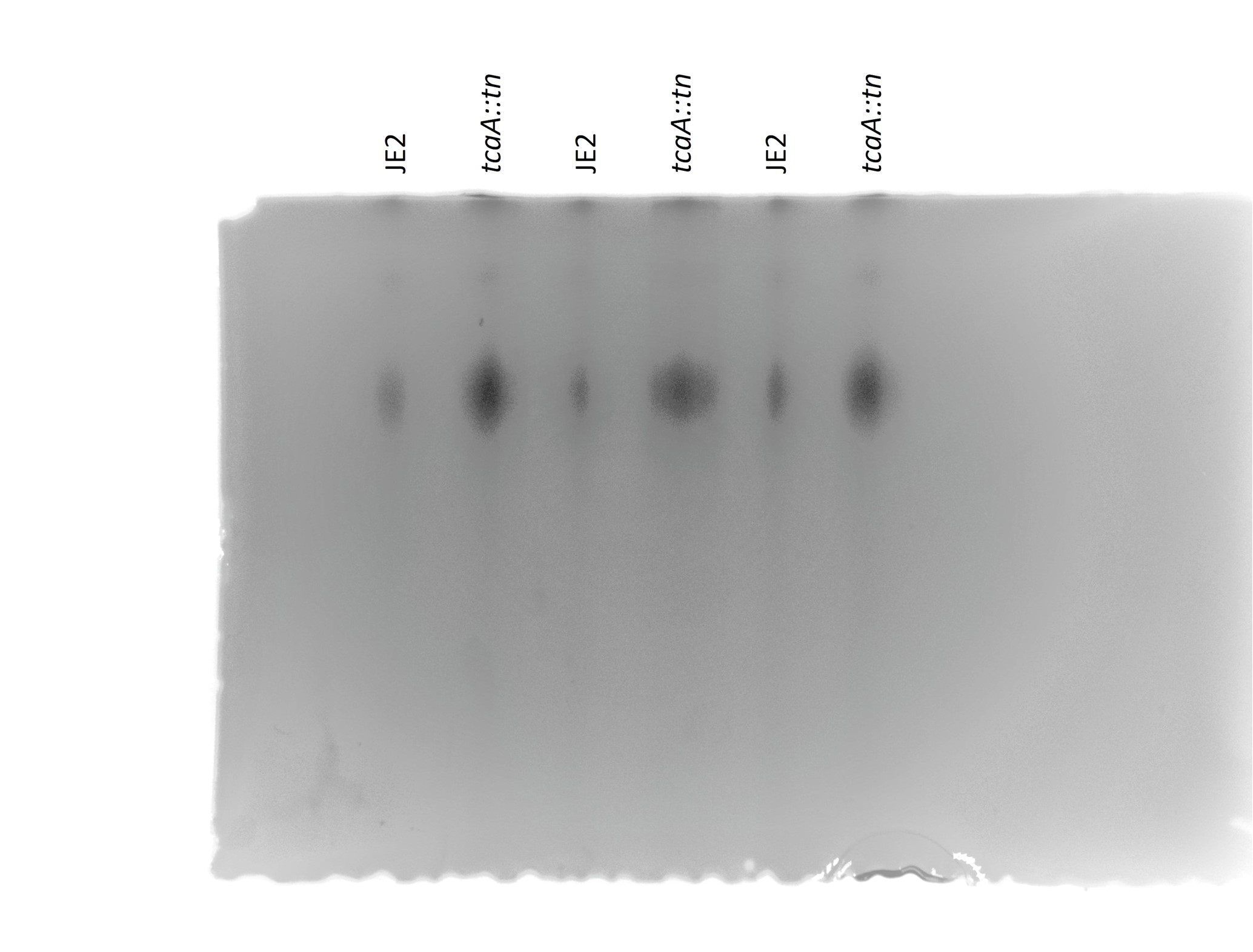

Supplement: Figure 5—source data 2. [file elife-87026-fig5-data2.docx]

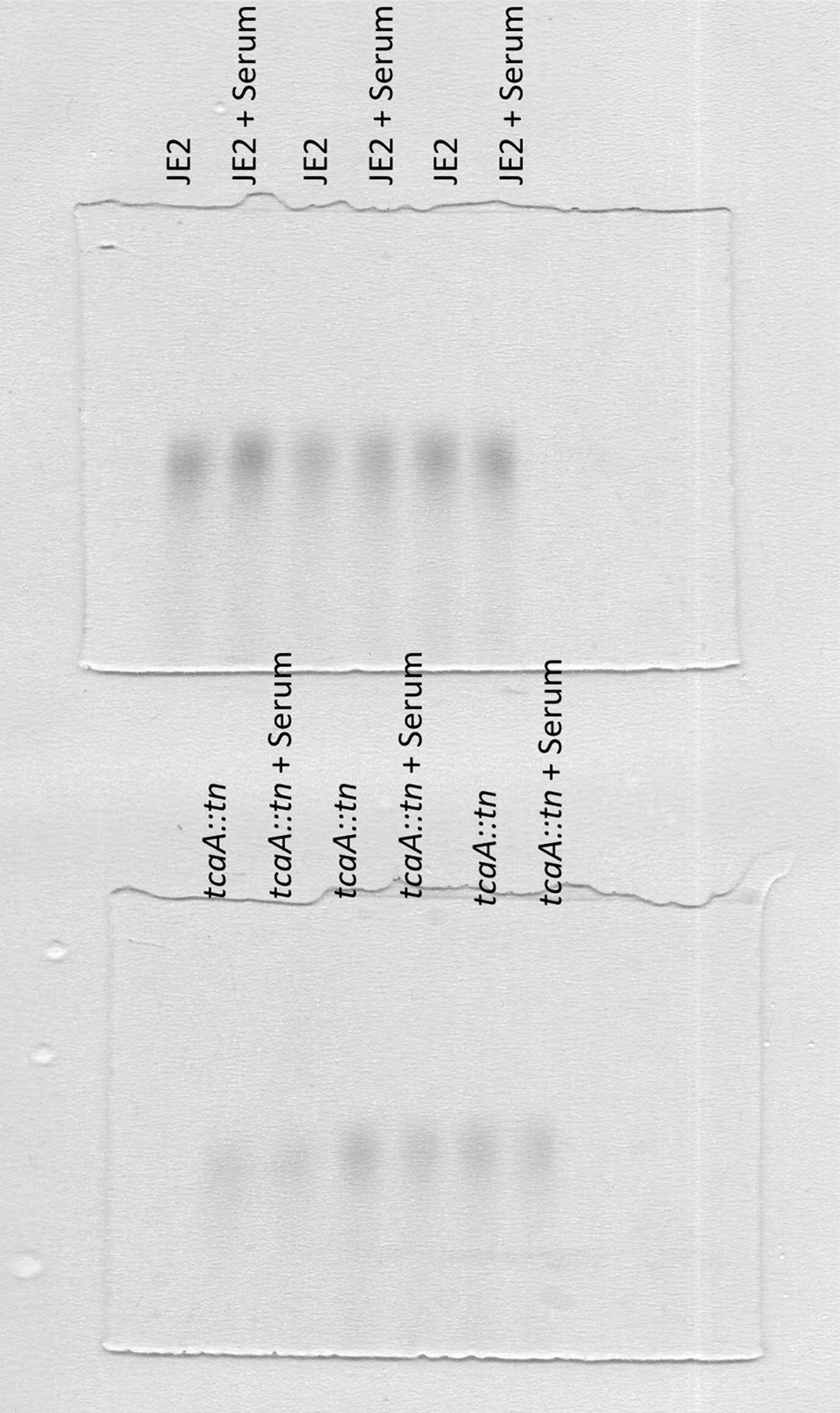

Supplement: Figure 6—source data 1. [file elife-87026-fig6-data1.docx]
